# Hypnosis: Adjunct Therapy for Cancer Pain Management

**DOI:** 10.6004/jadpro.2013.4.2.2

**Published:** 2013-03-01

**Authors:** Kathy Kravits

**Affiliations:** From City of Hope, Duarte, California

## Abstract

Pain is a symptom associated with prolonged recovery from illness and procedures, decreased quality of life, and increased health-care costs. While there have been advances in the management of cancer pain, there is a need for therapeutic strategies that complement pharmaceutical management without significantly contributing to the side-effect profile of these agents. Hypnosis provides a safe and efficacious supplement to pharmaceutical management of cancer pain. One barrier to the regular use of hypnosis is health-care providers’ lack of current knowledge of the efficacy and safety of hypnosis. Advanced practitioners who are well-informed about hypnosis have an opportunity to increase the treatment options for patients who are suffering with cancer pain by suggesting to the health-care team that hypnosis be incorporated into the plan of care. Integration of hypnosis into the standard of care will benefit patients, caregivers, and survivors by reducing pain and the suffering associated with it.

Pain is a symptom associated with prolonged recovery from illness and procedures, decreased quality of life, and increased health-care costs (NCCN, 2011; Montgomery et al., 2007; Green, Barabasz, Barrett, & Montgomery, 2005). It contributes to the suffering associated with cancer and its treatment. Although there have been advances in the management of cancer pain, there continues to be a need for therapeutic strategies that complement pharmaceutical management without significantly contributing to the side-effect profile of these agents. Hypnosis provides a safe and efficacious supplement to pharmaceutical management of cancer pain.

Research within the past 20 years has firmly established that hypnosis can be effective in addressing acute and chronic pain associated with cancer and its treatment (Montgomery et al., 2010; Lang et al., 2006; NIH, 1996). Hypnosis may be successfully used as a brief, time-limited adjunct to the primary pain management strategy. Side effects associated with hypnosis are rare. Despite these recognized and well-documented benefits, hypnosis has not been integrated into the standard of care for pain management of cancer patients.

One barrier to the regular use of hypnosis is many health-care providers’ lack of current knowledge of the efficacy and safety of hypnosis. Advanced practitioners make significant contributions to the management of cancer pain and are in a perfect position to support the use of hypnosis for adjunct pain management. The education and training of advanced practitioners in the use of hypnosis is essential for its successful integration into the standard of care for pain management of cancer patients. Although this article focuses on the role of the advanced practice nurse, it is recognized that physician assistants have also been trained in the practice of hypnosis.

## Definitions

The definition of hypnosis is controversial. Depending upon the theoretical framework embraced by a practitioner, hypnosis may be defined in a variety of ways. It has been defined by Division 30 of the American Psychological Association as "focused attention experienced by a receptive individual in response to an experience either facilitated by a hypnotist or self-guided. Suggestions are offered during the experience for changes in sensation, perception, cognition, affect, mood, or behavior" (Green et al., 2005). Another definition of hypnosis proposed by Montgomery and Schnur (2007) characterizes hypnosis as "an agreement between a person designated as the hypnotist (e.g., health-care professional) and a person designated as the client or patient to participate in a psychotherapeutic technique based on the hypnotist providing suggestions for changes in sensation, perception, cognition, affect, mood, or behavior." The significant difference between these two definitions is the emphasis that Montgomery and Schnur place on the relationship between hypnotist and client/patient and the identification of hypnosis as a psychotherapeutic technique. These differences are rooted in the theoretical orientation of the authors and do not define hypnosis using scientifically obtained data.

Suggestibility is the ability to respond to suggestions during a hypnosis experience. It is a quality common to most people and an important characteristic for the achievement of a successful hypnosis result (Kallio & Revonsuo, 2003; Crawford, 1994). The degree of suggestibility varies from person to person. There are indications that the mechanism of action of hypnosis is somewhat different in highly suggestible individuals than in those who are less suggestible (Kallio & Revonsuo, 2003). Clinically, most individuals are able to have a successful hypnosis experience (Taylor, Goehler, Galper, Innes, & Bourguignon, 2010).

## Procedural Elements

The techniques associated with creating a hypnosis experience are varied. One of the commonly employed techniques is initiated by an induction, followed by deepening and therapeutic suggestion, and concluded by reversal (see Table 1). It is usually facilitated by a hypnotist but may be self-delivered and/or facilitated by the use of aids such as prerecorded CDs. There is some evidence that prerecorded CDs are less effective than facilitated face-to-face hypnotic experiences (Askay, Patterson, & Sharar, 2009). The critical technical element that separates a hypnosis experience from a relaxation, guided imagery, or meditation experience is the use of therapeutic suggestion (Taylor et al., 2010). Individuals who have participated in hypnosis describe the experience in a variety of ways. Table 2 provides a list of some of the more common experiences.

**Table 1:**
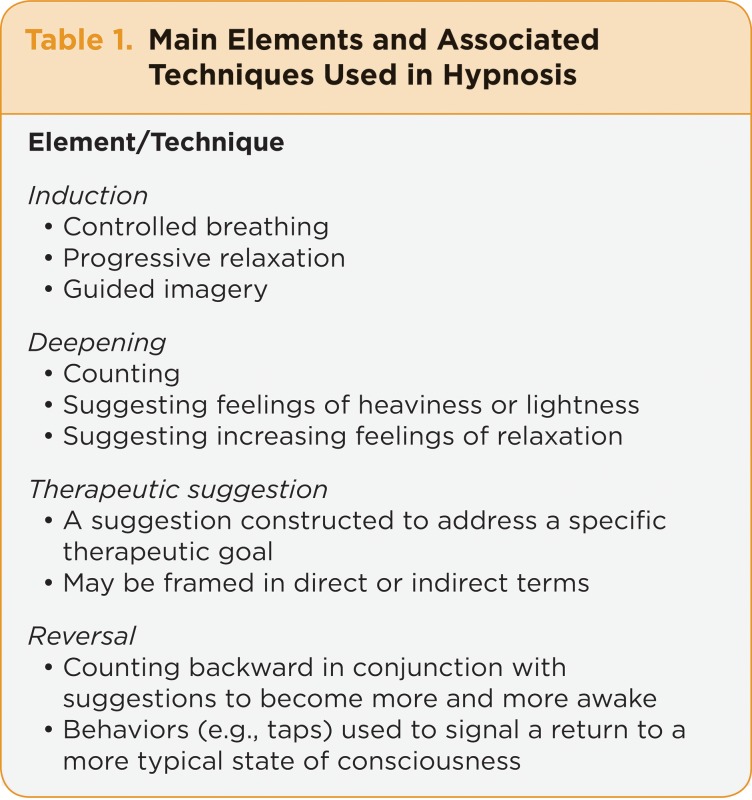
Table 1. Main Elements and Associated Techniques Used in Hypnosis

**Table 2:**
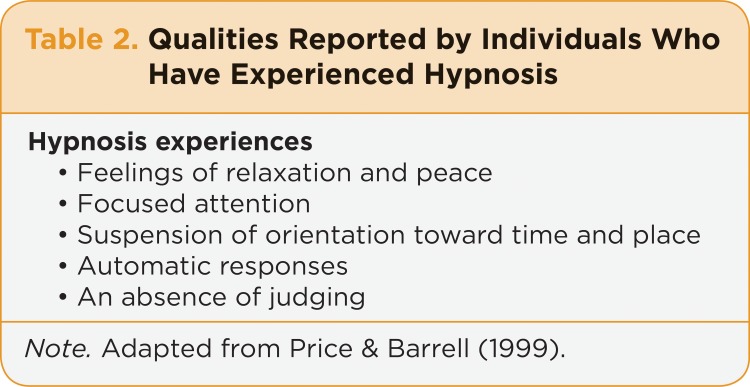
Table 2. Qualities Reported by Individuals Who Have Experienced Hypnosis

## Efficacy

There is significant evidence that hypnosis is effective at reducing cancer-related symptoms such as pain, nausea and vomiting, fatigue, and anxiety (Montgomery, David, Winkel, Silverstein, & Bovbjerg, 2002; Richardson et al., 2007; Lang et al., 2000; Lang et al., 2006; Neron & Stephenson, 2007; Elkins, Jensen, & Patterson, 2007; Montgomery et al., 2007; Schnur, Kafer, Marcus, & Montgomery, 2008; Jensen, 2009; Liossi, White, & Hatira, 2009; Mendoza & Capafons, 2009; Montgomery et al., 2010). In a seminal study by Montgomery and colleagues (2007), hypnosis was shown to decrease pain and anxiety postoperatively when administered preoperatively to patients undergoing breast cancer surgery. In the same study, it was demonstrated that health-care costs could be reduced by the administration of hypnosis. Montgomery’s findings corroborate the findings of Lang and colleagues reported in the *The Lancet*. Lang found hypnosis to provide effective procedural pain relief and those patients receiving hypnosis had shorter recovery times (Lang et al., 2000).

Hypnosis has been shown to be effective for use with adults and children. Liossi, White, and Hatira (2009) conducted a randomized trial comparing the effects of a lidocaine/prilocaine (EMLA) cream and hypnosis for pain control during venipuncture in pediatric patients between the ages of 7 and 16. They found that the patients who received hypnosis reported less pain, anxiety, and distress than those who received the cream.

It has been determined by the National Institute of Health Technology Assessment Panel that hypnosis is effective in alleviating chronic pain, including cancer pain, procedural pain, and nausea and vomiting (NIH, 1996). Hypnosis has been found to be not only effective in the management of procedural pain, anxiety, and distress but superior to structured attention, empathy, and IV analgesia for those conditions (Lang et al., 2006; Lang et al., 2008).

## Safety

Hypnosis is a safe technique when practiced by a well-trained, experienced, licensed health-care provider. Side effects associated with hypnosis reported in the literature are rare. One undesirable effect that may be encountered is the inadvertent retrieval of unpleasant memories (Lang & Laser, 2009). A well-trained hypnotist can anticipate this effect, assess for risk factors, and generate a hypnotic experience that minimizes the risk.

## Barriers to Use of Hypnosis For Symptom Management

Despite the preponderance of data, hypnosis for the management of cancer pain has not been incorporated into the standard of care in most institutions (Neron & Stephenson, 2007). Critical barriers to the incorporation of hypnosis into the standard of care for the management of cancer pain include (1) persistence of myths and misconceptions that cast hypnosis in a negative light among the public and the health-care community, (2) lack of understanding of the mechanism of action of hypnosis, and (3) limited patient access due to restricted availability of practitioners of medical hypnosis (Elkins, Jensen, and Patterson, 2007).

## MYTHS AND MISCONCEPTIONS

Many myths and misconceptions still persist regarding hypnosis. They create barriers to the use of hypnosis and its successful inclusion in standard practice. Examples of some of the myths associated with hypnosis are listed in Table 3. It is essential that the practitioner understand the client’s beliefs about hypnosis and engage in a collaborative dialog to respectfully dispel those beliefs that are inaccurate or unhelpful.

**Table 3:**
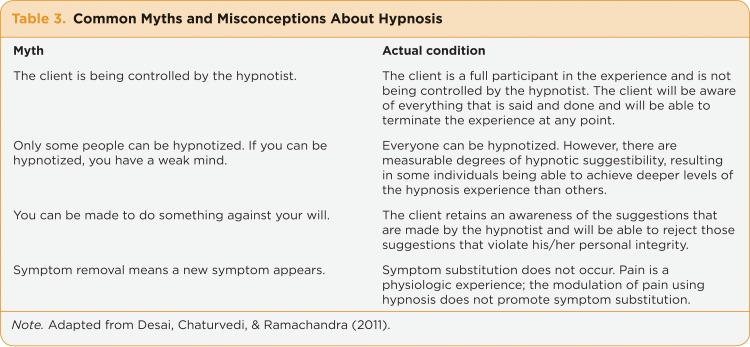
Table 3. Common Myths and Misconceptions About Hypnosis

## MECHANISM OF ACTION: CONTRIBUTIONS OF NEUROSCIENCE

With the advances in neuroscience in the past decade, a new understanding of the neural correlates of the hypnosis experience has been achieved. Two structures of the brain play a particularly important role in the hypnosis experience. They are the prefrontal cortex and the anterior cingulate cortex. These structures influence intentional physical self-regulation, cognitive control, and/or response to suggestions (Taylor et al., 2010). They also play a role in the integration of information relevant to ongoing social, cognitive, emotional, and psychological stress (Taylor et al., 2010). The prefrontal cortex serves as a critical structure in hypnosis due to its role in decision-making and its functional ability to influence autonomic nervous system activation.

The anterior cingulate cortex is located along the corpus callosum in the frontal lobe and influences cognitive functioning. It plays a significant role in attention and motivation. It is through the use of therapeutic suggestion that attention and motivation are focused on achieving desired outcomes.

Normally, there is a functional connection between the prefrontal cortex and the anterior cingulate that allows the prefrontal cortex to influence attention and motivation. During a hypnotic experience, there is a functional disconnection between these two structures, allowing attention to be more easily focused on achieving the therapeutic suggestions. There is a building body of evidence suggesting that this is the mechanism of action that allows hypnotic suggestion to produce a therapeutic effect (Taylor et al., 2010).

Research indicates that hypnosis modulates the pain experience and reduces perceptions of unpleasantness as well as pain intensity (Stoelb, Molton, Jensen, & Patterson, 2009; Jensen, Sherlin, Hakimian, & Fregni, 2008, 2009). Responses to hypnosis have been shown to be influenced by the expectations of the subject, suggestibility of the subject, phrasing of the induction and suggestions, and method of delivery (facilitated vs. recorded) of the induction (Barber, 2009; Gandhi & Oakley, 2005).

## LACK OF AVAILABLE PRACTITIONERS

The educational standards for medical hypnosis have traditionally been very restrictive. The professions most commonly eligible for training include physicians, psychoanalysts, dentists, and psychologists. This has changed within the past decade with the training of masters-prepared clinicians, including nurses (Lew, Kravits, Carberoglio, & Williams, 2011). The preparation of nurses to provide hypnosis would, if done on a wide scale at the time of graduate education, respond to the need for additional practitioners.

## Indications and Contraindications

Hypnosis is a safe and effective therapy for the management of many complex symptoms including but not limited to anxiety, distress, and pain. Hypnosis is indicated for use as an adjunct therapy for the management of cancer and cancer treatment–related pain. Those patients who may benefit from the addition of hypnosis to their symptom management regimens include those who experience dose-limiting side effects associated with opiate administration, elevated sedation levels associated with opiate administration, persistent problems with breakthrough pain, persistent anxiety directly related to anticipation of pain, and procedure-related pain. Hypnosis should not be used with patients who have a history of psychosis; a cognitive impairment that leads to an inability to concentrate; or personality disorders, particularly if psychotic features are attached (Deng et al., 2009).

## Implications for Advanced Practitioners

Advanced practitioners who are informed about the safety and efficacy of hypnosis have an opportunity to increase the treatment options for patients who are suffering with cancer pain by suggesting to the health-care team that hypnosis be incorporated into the plan of care. A goal of nursing practice is to reduce suffering. Integration of hypnosis into the standard of care for cancer pain management will benefit patients, family caregivers, and survivors by reducing pain and distress and the suffering associated with those symptoms. Advanced practitioners may play a vital role in the integration of hypnosis into the standard of care by establishing and influencing institutional philosophies and policies and serving as members of and advisors to important groups such as the Society of Psychological Hypnosis, the Society for Clinical and Experimental Hypnosis (SCEH), and the American Society of Clinical Hypnosis (ASCH).

## Future Directions

**RESEARCH**

Well-designed clinical trials of hypnosis are focusing on how to use the intervention as well as the nature of the hypnotic experience. Refinement of the timing of the administration of the intervention in relationship to treatment and procedures is being investigated. Identification of clinical populations and conditions that respond to hypnosis is another area of exploration that will provide useful information. Exploration of the feasibility of technology as a delivery vehicle for hypnosis will contribute to our understanding of potential strategies for increasing the availability of hypnosis to remote and underserved populations.

**EDUCATION OF NURSES**

In recent years, nurses have been identified as health-care providers who have the requisite education and experience to provide hypnosis. Increasing numbers of training programs for nurses are being sponsored by the leading national organizations (SCEH and ASCH). The standards for education of nurses to provide hypnosis as articulated by the SCEH are a minimum education of a master’s degree in a clinically oriented health-care field, license as a registered nurse, 40 hours of hypnosis training (16 hours of didactic and 24 of supervised clinical experience), and evidence of competence, which may be satisfied by membership in the SCEH.

Hypnosis is a nonlicensed practice. Professional organizations such as the SCEH, the ASCH, and the American Council of Hypnotist Examiners have taken it upon themselves to regulate the field and establish national standards for practice. It is important that individual practitioners become affiliated with one of these professional organizations to maintain practice standards and advance the use of medical hypnosis. The need for additional practitioners is recognized, and nurses have the opportunity to obtain the training necessary to provide this therapy.

## Conclusion

With the advent of systematic exploration of hypnosis by randomized controlled trials, a new understanding of its role in the management of disease- and treatment-related symptoms has been achieved. Advanced practitioners in oncology can make a significant contribution to advances in pain management by incorporating hypnosis into their practice and individual treatment plans.
